# Practical dilemmas and communication reconstruction of patients' right to informed consent under the Civil Code

**DOI:** 10.3389/fpubh.2026.1831405

**Published:** 2026-06-29

**Authors:** Junliang Chen, Luman Ye

**Affiliations:** 1 The First Affiliated Hospital of Chongqing Medical University, Chongqing, China; 2 Department of Big Data Engineering Center Children's Hospital of Chongqing Medical University, National Clinical Research Center for Children and Adolescents' Health and Diseases, Ministry of Education Key Laboratory of Child Development and Disorders, International Science and Technology Cooperation Base of Child Development and Critical Disorders, Intelligent Application of Big Data in Pediatrics Engineering Research Center of Chongqing Education Commission of China, Chongqing, China

**Keywords:** Informed consent, civil Code, reasonable-patient standard, therapeutic privilege, shared decision-making, health equity

## Abstract

China's Civil Code (2021) consolidates patients' right to informed consent and tightens the duty of disclosure owed by medical staff. In clinical practice, however, the substantive provisions of the Civil Code have not dissolved the persistent gap between statutory rule and everyday medical activity. This Policy and Practice Review diagnoses four interlocking practical dilemmas in the operation of the Code's informed-consent rule—covering defensive formalism, ambiguity over the decision-maker, the evidentiary architecture for consent and refusal, and the open-textured “inappropriate to explain” exception—and proposes a corresponding reconstruction along legal, cultural and technological dimensions, drawing on the reasonable-patient standard articulated in Canterbury v. Spence and Montgomery v. Lanarkshire Health Board (1, 2) and reaffirmed by the American Medical Association (3), the doctrine of therapeutic privilege as theorized by Faden and Beauchamp (4), and international guidance on informed-refusal documentation (5). By examining the institutional, cultural and technical factors contributing to these challenges, the article articulates implications for patient safety, equity for vulnerable populations, system-level trust and the dispute burden borne by clinicians and institutions, with a view to harmonizing legal compliance with humanistic, patient-centered care.

## 1 Introduction

Informed consent runs through the entire diagnosis and treatment process and is addressed by a layered Chinese legal framework comprising the Regulations on the Administration of Medical Institutions, the Practicing Physicians Law, the Regulations on the Handling of Medical Accidents, the Regulations on the Prevention and Handling of Medical Disputes, and the Civil Code. Yet practical difficulties persist. Internationally, by contrast, the field has progressively moved from a paternalistic-informative orientation toward deliberative and shared decision-making models (6, 7), and leading common-law jurisdictions and professional bodies have anchored the adequacy of disclosure to the informational needs of a reasonable person in the patient's position (1–3), while the doctrines of therapeutic privilege (4) and informed refusal (5) have been carefully circumscribed to prevent paternalistic erosion of patient autonomy. Set against this comparative background, this article analyses the practical difficulties facing patients' right to informed consent in Chinese medical practice and proposes a communication-based reconstruction with implications for patient safety, health equity and system-level trust.

Specifically, this Policy and Practice Review addresses three guiding questions. First, why have the substantive provisions on informed consent in the Civil Code (8, 9)—although doctrinally complete—failed to dissolve the persistent tension between rule and clinical reality? Second, in what respects does Chinese law lag behind, or diverge from, internationally established standards on the scope of disclosure, exceptions to disclosure, and documentation of patient refusal? Third, what concrete, multi-level reforms—legal, cultural and technological—can realign statutory rule with everyday clinical workflow without sacrificing patient autonomy or imposing unrealistic burdens on health professionals? In contrast to the chiefly doctrinal commentaries available in the existing Chinese literature (9–11), the contribution of this paper is twofold. Diagnostically, Section 5 distinguishes four interlocking practical dilemmas (D1–D4) under the Civil Code's informed-consent rule, grounding each in published empirical evidence from mainland Chinese clinical practice. Prescriptively, Section 6 proposes a reconstruction framework along legal, cultural and technological dimensions, with each subsection mapped directly to one of D1–D4. This explicit dilemma–reform pairing distinguishes the present analysis from earlier article-by-article doctrinal commentaries and aligns it with the audience of this journal.

## 2 Methods

This article is a narrative Policy and Practice Review combining doctrinal legal analysis with a synthesis of empirical evidence. Three categories of source were used. (i) Statutes, judicial interpretations and authoritative regulations: the Civil Code (2021); the Tort Liability Law (2009); the Practicing Physicians Law (1999, revised 2021); the Regulations on the Handling of Medical Accidents (2002); the Regulations on the Prevention and Handling of Medical Disputes (2018); and relevant Supreme People's Court interpretations. (ii) Doctrinal scholarship and empirical case studies, identified through searches of CNKI, Wanfang and PubMed (database-coverage years 2009–2025) using combinations of the Chinese terms 知情同意, 告知义务, 近亲属 and 医疗损害, and the English terms “informed consent,” “right to know,” “close relatives,” “therapeutic privilege” and “informed refusal.” Items were retained when they (a) addressed the operation of informed-consent rules in mainland Chinese clinical settings, (b) reported empirical case data or systematic doctrinal analysis, and (c) had been published in a peer-reviewed or core legal journal. Where possible, internationally indexed empirical studies of Chinese practice were preferred over Chinese-language doctrinal commentaries (12–15). (iii) International comparative materials, used in Section 6 to inform the proposed reconstruction: leading Anglo-American case law (Canterbury v. Spence; Montgomery v. Lanarkshire Health Board) (1, 2), professional guidance (American Medical Association Code of Medical Ethics; ACOG Committee Opinion 819) (3, 5) and the comparative legal and ethical literature on shared decision-making, therapeutic privilege and informed refusal (4, 6, 7, 16–21). Sources were synthesized under the three thematic dimensions used in the Reconstruction section (legal refinement, cultural reshaping, technological empowerment); illustrative empirical findings are summarized quantitatively where the original studies permitted.

## 3 Key conceptual clarifications and terminological conventions

Because the existing Chinese literature uses several closely related but legally distinct terms, this article adopts the following conventions throughout. (i) The “right to information” (知情权) denotes the patient's right to receive disclosure of diagnosis, treatment plan, risks, prognosis and alternatives during ordinary medical activities. (ii) The “right to informed consent” (知情同意权) is the more specific right, governed by Article 1,219 of the Civil Code (9), requiring explicit consent before surgery, special examinations and other high-risk interventions. (iii) Following the Supreme People's Court's interpretation, both rights are regarded as elements of a “special personal right” (特别人格权), the breach of which gives rise to non-pecuniary damages independent of any underlying physical harm (10, 22). The terms “close relatives” and “next of kin” appear interchangeably in earlier Chinese legal scholarship; for clarity, this article uses “close relatives” throughout, mirroring the wording of Article 1,045 of the Civil Code (23). The earlier “Tort Liability Law” (侵权责任法, 2009) is occasionally rendered “Injury Liability Law” in translation; the former is used here, consistent with the National People's Congress's official English translation.

Three further conceptual pairings recur below and warrant brief explanation. The “passive-active” doctor-patient model, sometimes also called the paternalistic model, captures relationships in which the patient's role is largely receptive while the physician decides; the “collaborative” or “shared decision-making” model, by contrast, treats the encounter as a two-way deliberation in which the patient's values and preferences are elicited and integrated with the physician's clinical judgment (6, 7). “Patient bodily sovereignty” is used in this article in the limited sense familiar from comparative bioethics: the moral and legal claim that decisions affecting a competent adult's own body belong, in the first instance, to that adult, not to the family or the institution (4). The Chinese clinical-classification scheme of Category I–IV surgeries (一类至四类手术) used by the National Health Commission grades procedures by technical complexity and risk, with Category IV designating the highest-risk procedures and “nationally restricted techniques” (限制类医疗技术) designating those requiring institutional accreditation and additional regulatory oversight.

## 4 Legislative development of the right to informed consent

### 4.1 Initial establishment phase

Since 1994, when the Regulations on the Administration of Medical Institutions were published, through the 1999 Practicing Physicians Law, to the 2002 Regulations on the Handling of Medical Accidents, Chinese laws have gradually addressed informed consent—but with notable limitations. The 1994 Regulations treated patient signatures as sufficient proof of consent, without specifying disclosure content or standards, and often prioritized family over patient consent, so that patient autonomy was not adequately respected. The Practicing Physicians Law promulgated in 1999 merely emphasized the obligation of cautious notification to avoid adverse effects on patients. The Medical Accident Handling Regulations promulgated in 2002 detailed the content of notification (such as the patient's condition, medical measures, and medical risks), but still focused on the unilateral obligation of notification and the avoidance of medical risks; the patient's right to consent was not independently emphasized. In public-health terms, this phase entrenched a paternalistic-defensive communication style: as long as a relative had signed, the institution treated its disclosure duty as discharged, and the patient's own comprehension of risks and alternatives was rarely measured or recorded (13, 14).

### 4.2 The stage of independent rights

In 2009, the Tort Liability Law for the first time established the independent status of patients' right to informed consent in civil law, incorporating this right into the infringement liability system. It also clarified the hierarchy of parties responsible for the duty of explanation: the patient themselves takes precedence, followed by close relatives, who may only be informed when it is inappropriate to explain to the patient directly. The content of explanations and forms of consent were also articulated across two tiers: first, for general medical activities with low risks, only the patient's condition and medical measures need to be explained, and the patient's right to consent is not emphasized; second, for higher-risk medical activities such as surgery and special examinations or treatments, not only must medical risks and alternative options be explained, but written consent from the patient is also required. If the patient is unable to give consent, the patient's close relatives may be consulted. The shift produced two visible health-system effects: (a) hospitals began to invest in standardized written consent forms—improving traceability but also seeding the formalism critiqued below—and (b) the proportion of medical-dispute claims explicitly grounded in alleged failure to inform began to rise (12).

### 4.3 Mature and well-established stage

Chapter VII “Tort Liability” in the Civil Code (8), which came into effect on 1 January 2021, is a comprehensive revision based on the original Tort Liability Law and related judicial interpretations. It inherits the essence of the original provisions while incorporating innovations consistent with the requirements of the new era. The patient's right to informed consent is no exception. Corresponding to the first sentence of Article 55 of the Tort Liability Law, the amendment changes “explain” to “specific explanation”, “written consent” to “explicit consent”, and “not suitable for explaining to the patient” to “cannot or should not explain to the patient” (9). These modifications require medical institutions and their staff to fulfill their notification obligations strictly—both formally and substantively. The introduction of “specific explanation” and “explicit consent” has strengthened and diversified the standards for fulfilling the duty of disclosure and the forms of consent, further highlighting the value implications of the patient's right to informed consent. From a public-health-systems perspective the 2021 reform was intended to push clinical practice from the “informative” model of the physician-patient relationship toward the “deliberative” and shared-decision-making models articulated by Emanuel and Charles and colleagues (6, 7), in which disclosure is the gateway to genuine partnership rather than a procedural precondition for surgery. Whether that aspiration has been realized in everyday practice is the empirical question to which the next section turns.

## 5 Practical challenges in the exercise of the right to informed consent

Although the Civil Code imposes stricter requirements on healthcare professionals' duty to inform than previous legislation, numerous challenges persist in clinical practice. Recent data indicate a rising proportion of patient claims against healthcare institutions for failure to fulfill, or improper fulfillment of, this duty (12). Three representative empirical studies of medical disputes adjudicated in mainland Chinese courts illustrate the scale and pattern of the problem. Li and colleagues analyzed 1,790 medical-damage liability litigation cases concluded between 2015 and 2021 in China and reported that compensation cases tended to increase year on year, with violation of informed-consent obligations among the recurrent grounds of liability (12). Xu and Yuan's 2024 questionnaire study of 368 young Chinese physicians documented a distinctive “doctor-family-patient” pattern in which physicians routinely informed family members rather than the competent patient, especially for older adults patients, and typically complied with family requests to withhold information (13). Chen and colleagues, conducting in-depth interviews with hospitalized patients in Guangdong, found that signing consent forms was widely understood as a hospital procedure rather than as the conclusion of a deliberation about risks and alternatives (15). Although none of these studies is a national probability sample, together they support the central empirical claim that the rule–practice gap is real, sizeable and patterned.

Drawing on these data and the doctrinal literature, this section identifies four interlocking practical dilemmas, summarized in Table 1: D1, defensive formalism without a substantive disclosure standard (section 5.1); D2, ambiguity over who exercises the right (section 5.2); D3, the limits of the evidentiary architecture for both consent and refusal (section 5.3); and D4, the absence of a bounded therapeutic-privilege doctrine (section 5.4). Each dilemma is matched to a corresponding reform proposal in Section 6.

**Table 1:** Four practical dilemmas in implementing the Civil Code's informed-consent provisions: legal roots, clinical manifestations and consequences.

Dilemma	Legal root	Clinical manifestation	Consequence
D1—Defensive formalism without a substantive disclosure standard (§5.1)	Article 1,219 specifies disclosure content but not the audience-centered yardstick by which adequacy is measured (9)	Departmental templates; brief reading-aloud of the form; signature obtained before genuine deliberation (15)	Erosion of patient autonomy; courts increasingly find disclosure inadequate despite signed forms (12)
D2—Ambiguity over who exercises the right (§5.2)	Civil Code lists patient → close relatives → institution but is silent on order among relatives or on limits to family override (23, 24)	Disclosure made to family by default; conflicts among relatives delay treatment; family decisions override the patient's expressed wishes (13)	Tragic outcomes (e.g., A parturient in Yulin requested cesarean delivery due to intolerable labor pain during vaginal birth, after the request was denied, the parturient jumped from a building and died.); rising litigation; loss of trust in the system (12, 14)
D3—Limits of the evidentiary architecture for consent and refusal (§5.3)	“Explicit consent” under Art. 1,219 is broader than “written consent” but lacks operational rules; no parallel rule for documenting refusal (9, 25)	Hospitals continue to rely on paper forms; oral/digital consent rarely usable as evidence; informed refusal not systematically documented	Unfair allocation of evidentiary burden; missed protection where patients refuse high-risk procedures
D4—Unbounded “inappropriate to explain”/therapeutic-privilege gap (§5.4)	Article 1,219 permits disclosure to relatives where it is “inappropriate” to explain to the patient, but the term is undefined and unconditioned (9, 26)	“Inappropriate” invoked broadly—e.g., older adults or anxious patients routinely bypassed; family preferences treated as clinical contraindication (13, 14)	Statutory exception expands into a paternalistic loophole; patient autonomy circumvented without clinical justification or procedural review

### 5.1 Defensive formalism and the absence of a substantive disclosure standard

Article 1,219 of the Civil Code requires healthcare professionals to give a “specific explanation” of medical risks and alternative treatment options (9). In practice, however, the obligation is frequently reduced to two formal acts: ensuring that a written consent form bears the patient's signature, and reading the form aloud or paraphrasing it cursorily before signing (15). Where the disclosure is templated, departmental forms list common or potential risks for a procedure type but rarely accommodate patients with atypical histories or specific clinical features—for example presenting identical risk language to patients with and without severe osteoporosis. Both behaviors—formalized signature collection and formulaic templated language—share a common root and a common consequence: courts increasingly find that the duty of disclosure was not fulfilled, even where the consent form is signed (10, 12, 22).

The doctrinal root of this defensive posture lies in two related features of the Civil Code. First, the provision is drafted as a duty owed by the clinician rather than as a right enabling the patient to actively seek information, so practitioners can avoid liability by staying within the formal boundaries of the listed disclosure items, without needing to verify whether the patient has actually understood (10). Second, and more fundamentally, the Civil Code lists what must be disclosed but does not identify the audience by reference to whose informational needs the adequacy of disclosure is to be measured. Without such a substantive yardstick, “specific explanation” defaults in practice to whatever the treating clinician judges sufficient, or to a fixed list dictated by professional habit—which is precisely how defensive formalism becomes the rational response to legal uncertainty (15). According to the Supreme People's Court's interpretation, the right to informed consent is itself a special personal right; failure to fulfill it adequately gives rise to non-pecuniary damages independent of any physical harm (22). Defensive formalism thus paradoxically increases legal exposure rather than reducing it. The remedy lies not in adding more items to the disclosure list, but in supplying the missing substantive standard—a task to which Section 6.1 returns.

### 5.2 Ambiguity over who exercises the right

Articles 1,219 and 1,220 of the Civil Code establish a three-tier hierarchy for receiving disclosure and giving consent: the patient themselves, the patient's close relatives, and—in emergencies where neither can be reached—the head of the medical institution or an authorized representative (9). Two structural ambiguities undermine the operation of this hierarchy in everyday practice. The first concerns the order of priority among close relatives; the second concerns the limits, if any, on family override of a competent patient's wishes.

Article 1,045 of the Civil Code defines close relatives broadly—spouses, parents, children, siblings, and grandparents and grandchildren of either line (23). No statute or judicial interpretation specifies the order of precedence among them, and disagreements among relatives about treatment are inevitable. Two academic positions have emerged: one proposes a statutory order tracking inheritance succession, with spouses, children and parents as first priority and the remaining categories ranked behind (24); the other follows guardianship hierarchy, in which spouses precede parents and children, and other close relatives come third. Both proposals presuppose, however, that family decision-making remains the default once the patient cannot personally consent. This default itself is open to challenge where a relative's decision conflicts with the patient's prior expressed wishes or with the patient's best interests (16, 17).

The deeper problem is that, in practice, healthcare institutions often fulfill the duty of disclosure to relatives even where the patient is competent, conscious and capable of understanding the explanation. Empirical studies of Chinese clinical practice consistently document this pattern: in Xu and Yuan's 2024 questionnaire of young physicians, the patient's family was treated as the primary or co-equal informational audience in a majority of scenarios, particularly for older adults patients (13). Raposo's comparative analysis similarly observes that Chinese implementation of informed consent frequently routes disclosure through the family in ways that diverge from the autonomy-centered model assumed by the formal legal text (14). The 2017 Yulin maternal tragedy —in which a 26-year-old woman in protracted labor at the First Hospital of Yulin in Shaanxi Province repeatedly requested a cesarean section over an extended period; the hospital had earlier required a written authorization transferring her decision-making power to her husband; her family declined to consent to the surgery; and the patient ultimately fell from a fifth-floor window of the labor ward (14)— exemplifies the worst-case manifestation of this pattern: a competent adult's own decision was effectively subordinated to a family veto operating through an institutional consent procedure. The case is extreme, but the underlying mechanism—disclosure and consent routed through relatives by default—is documented across more routine clinical settings (13, 15).

### 5.3 Limits of the evidentiary architecture for consent and refusal

The 2009 Tort Liability Law required “written consent” for high-risk procedures, principally for evidentiary reasons: written documents are fixed, traceable and judicially recognized. The Civil Code replaces “written consent” with “explicit consent,” recognizing that consent may also be conveyed orally, by audio-visual recording, or through text-based digital channels (9, 25). In practice, however, hospitals continue to rely almost exclusively on paper forms (12). Three factors explain the persistence: legal-risk prevention, since the medical institution must prove that it has fulfilled its disclosure obligations and the written consent form, owing to its fixity and judicial recognition, remains the most direct form of evidence, while oral or audio-visual material faces tampering and integrity challenges; practice-oriented inertia, since after more than a decade under the Tort Liability Law institutions developed mature templates, signing procedures and archives, and in the absence of clear operational guidelines for non-written modalities most institutions have continued the established practice; and technical and cost bottlenecks, since blockchain evidence storage, AI-based voice transcription and the long-term retention of audio-visual records on dedicated servers all impose substantial cost, primary-care facilities cannot easily afford them, and judicial use of digital evidence often requires notarisation that lengthens dispute resolution (25).

A symmetrical evidentiary problem, but one that has received considerably less attention in Chinese practice, concerns informed refusal. The Civil Code and its supporting regulations devote detailed attention to documenting consent, yet contain no equivalent procedural rule for the situation in which a competent patient (or, where applicable, their close relatives) declines a recommended intervention. At present, when a patient refuses a recommended high-risk procedure and an adverse outcome ensues, hospitals frequently struggle to demonstrate that adequate disclosure preceded the refusal: there is no standardized refusal-of-treatment form, no requirement to record the patient's reasons or capacity assessment, and no rule on witnessing such a decision. This evidentiary asymmetry between consent and refusal is itself a structural feature of the current architecture, one that allocates the evidentiary burden unfairly and leaves the patient's right to refuse less well protected than the right to consent.

### 5.4 Unbounded “inappropriate to explain”: the therapeutic-privilege gap

Article 1,219 already permits a clinician not to explain directly to the patient where it is “inappropriate” to do so, in which case the duty is discharged to close relatives (9). The provision is, however, almost devoid of operational content: “inappropriate” is undefined, no conditions are placed on its invocation, and no procedural safeguards exist against its over-use. Domestic doctrinal commentary (26) characterizes “inappropriate to explain” as covering situations in which, given factors such as illness, age, personality, cognitive status and psychological resilience, informing the patient may cause adverse psychological effects. The standard so framed is open-textured: each of these factors is itself a matter of clinical judgment, and the provision does not require the judgment to be documented, peer-reviewed or time-limited.

In clinical practice the gap is consequential. Empirical studies suggest that the exception is invoked broadly: in Xu and Yuan's survey, when family members requested that physicians withhold information from patients “in the best interest of the patient,” the majority of physicians complied even though many reported personal moral discomfort with doing so (13). Comparative analyses of Chinese informed-consent practice document parallel patterns of family-induced withholding for older adults or anxious patients (14). None of these is a clinical contraindication to disclosure in the strict sense; each is, in effect, a reframing of family preference or institutional risk-management as a therapeutic justification. The structural problem is that the Civil Code provides the exception without the discipline: it lists when the duty migrates from patient to family, but does not specify the harm that must be foreseeable, the documentation that must be made, or the patient's residual right to information on request. Without such bounds, an exception intended for genuinely exceptional cases expands into a generalized paternalistic loophole, and the autonomy gains of Article 1,219 are eroded at the bedside. Section 6.1 returns to this dilemma and proposes a tightly circumscribed therapeutic-privilege doctrine to fill the gap.

## 6 Reconstructing communication regarding the right to informed consent

Based on the foregoing analysis, although the provisions on the right to informed consent in medical damage liability under the Civil Code have become relatively complete since its entry into force, and are of great significance for protecting the legitimate rights and interests of patients and balancing the doctor-patient relationship, practical difficulties remain. With the transformation of the doctor-patient relationship model toward a patient-centered orientation (6, 11), the value concepts and institutional functions embodied in the Civil Code's provisions have not been fully exerted. This article, on the basis of concretising the doctor-patient relationship, life scenarios and social facts, undertakes a communication-based reconstruction of the right to informed consent from multiple dimensions—improvement of the legal system, cultural reshaping, and technological empowerment—so as to better conform to the essential connotation of the right to informed consent and exert its value concepts and institutional functions.

**Figure 1:**
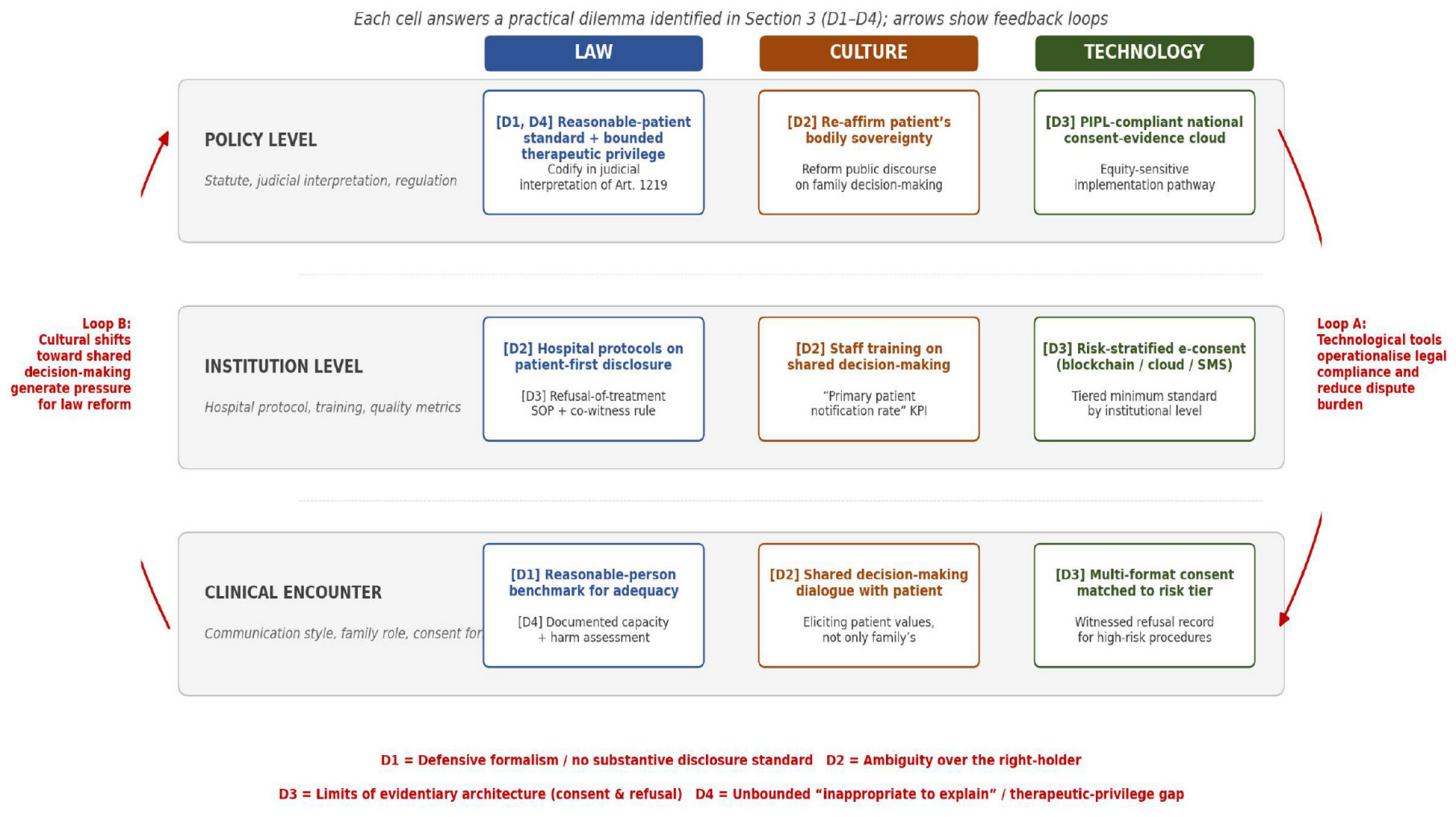
Three-dimensional reconstruction framework for the right to informed consent: law, culture and technology, operating across policy, institution and clinical-encounter levels. Each matrix cell is tagged with the dilemma(s) (D1–D4) it answers; two red feedback loops connect the levels (A: technology operationalises legal compliance; B: cultural shifts pressure law reform).

Figure 1 summarizes the proposed reconstruction and its mapping to the four dilemmas identified in Section 5 (D1–D4). Three substantive dimensions—law, culture and technology—operate across three system levels: policy (statute, judicial interpretation and regulation), institution (hospital protocol, training and quality metrics) and clinical encounter (communication style, family role and consent format). Each cell of the matrix is labeled with the dilemma(s) it addresses, so that the reader can trace each reform back to a concrete problem. Two feedback loops are central. Loop A runs downward: technological tools (blockchain consent platforms, regional cloud-evidence systems, risk-stratified e-consent templates) operationalise legal compliance and reduce the dispute burden on clinicians. Loop B runs upward: cultural shifts toward shared decision-making, accumulating in everyday clinical encounters and institutional training, generate political and professional pressure for further legal refinement (7). Without Loop A, legal reform remains inert at the bedside; without Loop B, technology and law drift apart from social legitimacy.

### 6.1 Improve the legal system

The shift from the Tort Liability Law to the Civil Code in framing informed-consent rights not only responds to past practice and academic discussion but also demonstrates legislative innovation in improving the protection mechanism of patients' informed consent rights (8, 9). However, with the continuous development of the economy and society and the continuous advancement of law-based governance, the change in existing rules does not mean that they are universally applicable to all real situations. It is still necessary to continuously refine and improve potential legal issues in practice. This article therefore proposes targeted improvements to the relevant legal system in order to address dilemmas D1–D4 identified in Section 5.

#### 6.1.1 Anchor “specific explanation” in a reasonable-patient standard (addresses D1)

After the implementation of the Civil Code, medical disputes arising from the right of informed consent have not been effectively alleviated. This stems in part from the Civil Code's provisions perpetuating the traditional passive-active doctor-patient relationship model, which places greater emphasis on the statutory duty of healthcare providers to fulfill their obligation to inform proactively, while failing to safeguard patients‘ right to actively seek medical information (10, 15). The existing legal framework must therefore not only require healthcare professionals to diligently fulfill their statutory duty to inform but also safeguard patients' right to proactively access treatment information, gradually transforming the passive-active relationship into a collaborative model and enabling more prudent decision-making (6, 7).

Equally important, the dilemma identified in section 5.1—the absence of a substantive yardstick for adequate disclosure—can be resolved only by importing such a yardstick into the operation of Article 1,219. Comparative experience strongly supports a reasonable-patient (or material-risk) standard. In Canterbury v. Spence (464 F.2d 772, D.C. Cir. 1972), the United States Court of Appeals rejected the “professional custom” test and held that the scope of disclosure should be measured by what a reasonable person in the patient's position would consider material in deciding whether to undergo the proposed treatment (1). The United Kingdom Supreme Court reached a substantively identical conclusion in Montgomery v. Lanarkshire Health Board [2015] UKSC 11, replacing the prudent-doctor (Bolam) test with a duty to ensure that the patient is aware of any material risks, materiality being assessed from the perspective of a reasonable person in the patient's position, supplemented by attention to risks the particular patient would be likely to attach significance to (2). The American Medical Association's Code of Medical Ethics articulates the same orientation, stressing that the physician's duty is to provide patients with the information they need to make well-considered decisions about their care, not the information the physician believes they ought to receive (3). The doctrinal foundation for this audience-centered test was elaborated by Faden and Beauchamp in their canonical history and theory of informed consent, which traces the legal and ethical convergence on a patient-oriented standard (4). We therefore propose that judicial interpretation of Article 1,219—whether through Supreme People's Court guidance or relevant administrative regulations—make explicit that the adequacy of “specific explanation” is to be assessed by what a reasonable person in the patient's position would consider material to the decision, with attention to particular concerns the clinician knew or ought reasonably to have known the patient held. Anchoring “specific explanation” to this objective benchmark would limit defensive formalism, restore disclosure to its rightful function as a precondition of meaningful autonomy, and provide adjudicators with a workable test for evaluating breach.

#### 6.1.2 Clarify the order of succession and limits of substitute consent (addresses D2)

As individuals sharing a close blood relationship with the patient, close relatives typically possess detailed familiarity with the patient's living circumstances and physical condition. When the patient is unable or unsuited to exercise their right to informed consent, close relatives are often best positioned to decide from the patient's perspective. Consequently, the Civil Code designates close relatives as second-priority decision-makers after the patient themselves, though it does not specify the order of precedence among them (23). In practice, excessively broadening the scope of close relatives or treating all relatives' opinions equally would not only increase the burden on medical institutions but, more critically, delay the patient's optimal treatment window. Therefore, unless explicitly authorized by the patient, a hierarchical order should govern the exercise of substitute informed consent. Two prevailing academic views exist (24): one proposes a statutory order of succession, ranking spouses, children and parents as first priority, followed by siblings, paternal and maternal grandparents; the alternative follows guardianship hierarchy: for minors, parents are first priority; for adults, spouses take precedence, followed by parents and children, with third priority accorded to other close relatives. Where explicit authorization from the patient is absent and conflicting opinions arise among relatives of differing priority, the opinion of the first-priority relative shall prevail. To safeguard the patient's legitimate rights and interests, restrictions should be imposed on substitute consent. Should a close relative's decision contravene the patient's best interests or even severely jeopardize their right to life and health, the healthcare institution must reject that opinion (16, 17, 27) and proceed with standard medical practices in accordance with the patient's interests.

#### 6.1.3 Codify a bounded therapeutic privilege (addresses D4)

The therapeutic-privilege gap identified in section 5.4 is best closed by developing the existing “inappropriate to explain” language of Article 1,219, through subordinate legislation or judicial interpretation, into a clearly bounded doctrine along the lines recognized in many jurisdictions (19). Therapeutic privilege permits a physician to withhold specific information from a competent patient where, in the physician's reasonable clinical judgment, full disclosure would itself cause serious psychological harm or significantly worsen the patient's prognosis (20). It is an exception to, not an abrogation of, the duty to inform. The doctrine has been recognized as part of Polish health law under the Act on the Professions of Physician and Dentist and the Act on Patients' Rights and the Patient Ombudsman, both of which permit limited withholding of information where disclosure would be detrimental to the patient, while reaffirming that the patient retains the right to know the diagnosis on request (21).

Three safeguards should accompany any Chinese codification. First, the privilege must never extend to complete concealment of the diagnosis when the patient asks; the right to be informed on request is non-derogable (3). Second, withholding must be justified by serious, concrete and clinically foreseeable harm flowing from the disclosure itself, not by the physician's prediction that the patient might decline beneficial treatment if fully informed—a misuse the AMA Code of Medical Ethics and the Canterbury court both expressly rejected (1, 3). Third, where the privilege is invoked, disclosure should ordinarily be made to a close relative, the reasoning recorded in the medical record, and the privilege's continued operation periodically reassessed (19). Codifying these conditions would discipline the existing “inappropriate to explain” language of Article 1,219, prevent its expansion into a generalized paternalistic loophole, and align Chinese law with internationally accepted ethical and legal practice.

### 6.2 Cultural reimagining

Tracing the legislative evolution of informed consent rights reveals that China's healthcare decision-making framework (11) has undergone a systemic transition: from family members or associates holding primary authority, to shared decision-making between patients and their families, and finally to a patient-centered approach prioritizing the patient's role. This progression reflects legislators' respect for patient dignity while establishing a safeguarding system centered on protecting patient rights. Nevertheless, influenced by traditional Chinese family values, many medical decisions continue to be made primarily through joint patient-relative deliberation—or even solely by relatives—despite patients possessing full legal capacity (13, 14). The cultural strand of the reconstruction therefore complements the legal strand of section 6.1 by addressing the same dilemma (D2) at the level of clinical and institutional norms.

It is helpful to situate this Chinese trajectory within comparative bioethics. Substantively similar problems—how to balance respect for autonomy against family involvement, how to determine “best interests” in incapacitated patients, how to operationalise shared decision-making—have been addressed in the Anglo-American literature for decades (4). Coggon's analysis of “best interests” and Taylor's critical evaluation of best-interests decision-making both insist that the assessment cannot be reduced to medical judgment alone but must integrate the patient's own values (16, 17). Charles, Gafni and Whelan's influential framework treats shared decision-making as a stepwise process of information exchange, deliberation and joint decision, in which the family is one informational input among others rather than a substitute decision-maker (7). Emanuel and Emanuel's typology distinguishes paternalistic, informative, interpretive and deliberative models, with the deliberative model approximating the partnership envisaged by Article 1,219 (6). The Chinese reforms proposed below should therefore be read not as importing a foreign framework, but as supplying culturally appropriate operational tools for a model of decision-making toward which Chinese statutory law has itself been steadily moving.

#### 6.2.1 Firmly establish the fundamental principle of putting patients first

The patient's autonomous decision-making authority holds absolute statutory precedence under the Civil Code's hierarchy (9). Substitute decision-making by close relatives may only be invoked when objectively impossible (e.g., unconsciousness) or subjectively inappropriate (e.g., implementing protective medical measures, now to be circumscribed under the therapeutic-privilege doctrine of section 6.1.3). Practice reveals numerous instances in which substitute decision-making has severely compromised patients' fundamental rights to life and health, as illustrated by the Yulin maternity tragedy described in section 5.2 (14). To progressively dismantle China's cultural inertia of family-based substitute decision-making, a three-tier safeguard mechanism should be established, so as to support a cultural shift from “patriarchal family authority” to “patient bodily sovereignty” (4). First, establish a patient decision-making capacity assessment process to prevent the circumvention of autonomy on grounds such as age or illness. Second, prioritize the validity of advance medical directives and the patient's wishes expressed while lucid, to counteract overstepping interventions by close relatives under the guise of “acting in the patient's best interests” (16, 17). Third, systematically retrain healthcare professionals to overcome the entrenched practice of prioritizing close relatives as primary recipients of information (13), while considering the incorporation of “primary patient notification rates” into clinical performance metrics.

#### 6.2.2 Carefully determine the specific circumstances for exercising the right of subrogation

Where it is “impossible or inappropriate to explain the patient's condition to them personally,” close relatives must exercise the right to informed consent on their behalf (9). To safeguard the statutory primacy of the patient's right, healthcare professionals must rigorously and prudently interpret these circumstances. “Unable to explain to the patient” typically denotes that the patient lacks capacity to consent (26). “Inappropriate to explain to the patient,” as developed in section 6.1.3 above, should be conceptualized as the Chinese counterpart of therapeutic privilege and accordingly tightly constrained. As substitute exercise of informed consent by close relatives directly affects the patient's autonomous decision-making rights, the applicable circumstances should be carefully assessed across multiple dimensions. First, establish a typological definition table to translate ambiguous “unable or unsuited” scenarios into operational clinical criteria. Second, develop a dual-review procedure for substitute decision-making based on “medical necessity + ethical appropriateness.” Third, introduce a judicial emergency adjudication mechanism for substitute conflicts (e.g., the Medical Dispute Expedited Adjudication Panel of Shanghai Pudong New Area Court, which issues rulings on substitute efficacy within 24 h).

### 6.3 Technological empowerment

Although the Civil Code has changed the method of informed consent from “written consent” to “explicit consent” (9, 25), medical institutions, owing to factors such as judicial validity, technology and cost, still mostly adopt written consent—the dilemma identified in section 5.3 (D3). To break this practical reliance and promote full-cycle evidence management of multiple forms of informed consent and refusal, systematic construction can be undertaken at different levels from the perspective of technological empowerment, and flexibly implemented according to differing circumstances.

#### 6.3.1 Implement differentiated technical solutions according to healthcare institution tiers

Significant disparities exist in information technology capability, management standards, and financial resources across healthcare institutions of varying tiers. Differentiated technical adaptation must therefore be adopted by tier. For tertiary institutions, prioritize deployment of a “blockchain + AI evidence preservation” platform, enabling real-time consolidation of multi-format consent evidence including audio/video recordings of consultations and electronic signatures. For secondary institutions, integration with the “Regional Medical Cloud Evidence System” is recommended; this enables centralized storage and one-click retrieval of audio-visual recordings via shared cloud servers within medical consortia. Primary care facilities may use the “Informed Consent Cloud” centrally developed by health authorities, which provides basic services such as SMS evidence storage and conversion of phone-call transcripts into text. International evidence supports tiered implementation: a 2021 systematic review of digital tools for informed consent reported consistent gains in patient comprehension and satisfaction across multimedia, web-based and tablet platforms, while flagging the need for offline compatibility and integration with existing clinical workflows in lower-resource settings (28).

#### 6.3.2 Different forms of informed consent should be adopted according to different circumstances

Although technological empowerment enables the evolution from traditional written informed consent to multi-format approaches, healthcare professionals' demanding workloads and the greater complexity of alternative methods necessitate a pragmatic approach. Rather than adopting multiple formats for their own sake, a dynamic selection mechanism should be established based on risk classification and format matching. For low-risk Category I and II procedures, a simplified written notification format may be employed. For medium-risk Category III procedures, personalized written notifications should be tailored to the patient's individual characteristics, supplemented where necessary by audio or video recording for evidentiary purposes (28). For high-risk procedures (e.g., Level 4 surgeries, nationally restricted techniques) and vulnerable populations (e.g., illiterate individuals, older adults patients, potential dispute risks), a multi-dimensional approach comprising “video explanation + audio recording + witnessing by close relatives” must be employed, with blockchain-based evidence storage ensuring full process traceability. Beyond this three-tier classification, an emergency-treatment green channel should be established. In life-threatening emergencies, consent may be obtained via instant communication tools such as telephone recordings or video calls, with the system uploading records in real time to the corresponding cloud platform.

#### 6.3.3 Mandate written documentation of refusal of treatment, with a second clinician as witness for high-risk cases (addresses D3)

The risk-stratified architecture above governs how consent is captured. A symmetrical architecture is required for the parallel scenario, identified in section 5.3, in which a competent patient (or, where applicable, lawful proxy decision-makers) refuses a recommended intervention. International guidance is well developed: professional bodies such as the American College of Obstetricians and Gynecologists and the American Medical Association recommend that an informed refusal be documented as carefully as informed consent, recording the nature of the recommended treatment, the risks of forgoing it, the patient's reasons, and confirmation of the patient's decision-making capacity (3, 5). Hospital risk-management literature in common-law jurisdictions further suggests that, where feasible, the refusal be reduced to writing on a dedicated “refusal of treatment” form, that the conversation be entered contemporaneously into the medical record, and—particularly for high-risk procedures—that a second healthcare professional witness and counter-sign the refusal entry (18).

We therefore propose that Chinese healthcare institutions adopt a standardized “refusal of treatment” form for any procedure carrying material clinical risk, in which the medical record explicitly documents (i) the patient's diagnosis, (ii) the recommended treatment, (iii) the disclosed risks of forgoing it, (iv) the patient's stated reasons for refusal, (v) confirmation that the patient's decision-making capacity has been clinically assessed, and (vi) the patient's signature (5, 18). For patients who are unable or unwilling to sign, a contemporaneous narrative entry attested by the responsible clinician should be entered, and the underlying refusal preserved through the same multi-format evidence channels (audio recording, video, time-stamped electronic note) used for high-risk consent. Critically, for refusal of high-risk procedures (Level 3 and Level 4 surgeries, nationally restricted techniques, refusal of life-saving emergency interventions), a second healthcare professional should be required to witness and counter-sign the refusal entry (18). Co-witnessing both protects the patient—by introducing a second clinical perspective at the point of a consequential decision—and protects the institution evidentially. Embedding informed-refusal documentation within the same regional cloud-evidence platform that holds the corresponding consent records would close the long-standing evidentiary asymmetry diagnosed in section 5.3 and ensure that the patient's right to refuse, no less than the right to consent, is fully and verifiably honored.

#### 6.3.4 Feasibility, privacy, and equity considerations for the technological proposals

The technological proposals above carry real implementation costs and risks that should be acknowledged rather than glossed. Three issues are particularly salient. (i) Data protection and privacy. Audio-visual consent recordings, biometric signatures and AI voice-transcript indices are sensitive personal information within the meaning of China's Personal Information Protection Law (29). Any consent-evidence platform must therefore satisfy the PIPL's requirements on separate consent for sensitive data, purpose limitation and minimum-necessary processing, in addition to the existing rules on medical-record confidentiality. Without an explicit governance layer, an evidentiary improvement risks creating a new privacy liability. (ii) Inter-institutional governance and interoperability. Regional cloud-evidence systems and a national “Informed Consent Cloud” require shared technical standards, common data dictionaries and clearly allocated custodial responsibilities; otherwise, evidence captured in one institution may be inadmissible or unreadable in another (28). (iii) The digital divide. Tertiary urban hospitals can absorb blockchain-based platforms, but primary-care facilities and rural township hospitals—which serve disproportionate numbers of older adults, illiterate and low-income patients—cannot. Imposing the same technical regime uniformly would deepen, rather than narrow, existing health inequities, a risk amply documented in international evaluations of consent interventions among low-literacy and disadvantaged populations (30, 31).

To make the proposals realistic rather than aspirational, an incremental implementation pathway is appropriate: regional pilot projects with central evaluation against pre-specified metrics; a tiered minimum standard, with primary-care facilities required only to implement low-cost text-and-voice components; and a national evaluation framework that disaggregates results by patient age, literacy and rural/urban setting, so that any equity gap is detected and remediated early (30, 31).

## 7 Prioritization of reforms: short-, medium- and long-term actions

Drawing the legal, cultural and technological strands of Section 6 together, this section sets out a workable agenda for sequenced implementation. Table 2 summarizes the proposed reforms across all three dimensions by time horizon and responsible actor, providing a roadmap for legislators, judicial bodies, hospital administrators, professional associations and IT vendors.

**Table 2:** Prioritization of proposed reforms by time horizon and responsible actor.

Time horizon	Reform	Primary responsible actor (s)
Short-term (1–2 years)	Hospital protocols requiring patient-first notification; standardized capacity-assessment SOPs; standardized refusal-of-treatment forms; basic e-consent (SMS, time-stamped EHR notes) in primary care	Hospital administrators; professional associations (CMDA, CMA); National Health Commission technical guidance
Medium-term (3–5 years)	Judicial interpretation anchoring “specific explanation” in the reasonable-patient standard; codification of therapeutic privilege with three safeguards; co-witness rule for refusal of Level 3–4 procedures; regional medical cloud-evidence pilots	Supreme People's Court; National Health Commission; provincial health bureaus; IT vendors
Long-term (5–10 years)	Statutory clarification of close-relatives hierarchy; national “Informed Consent Cloud” with PIPL-compliant governance; emergency judicial-adjudication mechanism for substitute conflicts; national evaluation framework disaggregated by age, literacy and rural/urban setting	Standing Committee of the National People's Congress; State Council; National Health Commission; Ministry of Justice

## 8 Conclusion

Continuously refining the practice of informed consent through diversified methods can better protect the legitimate rights and interests of doctors and patients, promote mutual understanding and trust, and thereby help build a harmonious doctor-patient relationship while reducing medical disputes. The reconstruction proposed here advances four concrete priorities, matched to the four dilemmas diagnosed in Section 5. First, the substantive standard of “specific explanation” under Article 1,219 should be aligned with the internationally established reasonable-patient (or material-risk) test, so that adequacy of disclosure is measured against the informational needs of a reasonable person in the patient's position rather than the discretion or habit of the treating clinician (D1) (1–3). Second, the order of precedence among close relatives and the limits of family override should be clarified, restoring the statutory primacy of the patient (D2) (16, 17, 24, 27). Third, the existing emphasis on documenting consent should be matched by an equally rigorous regime for documenting informed refusal, including written refusal forms and, for high-risk procedures, co-signature by a second clinician (D3) (5, 18). Fourth, the Civil Code's open-textured “inappropriate to explain” language should be developed into a tightly circumscribed therapeutic privilege, available only on documented clinical grounds, never used to override a patient's express request for information, and always coupled with disclosure to a close relative (D4) (19–21). Together with the law–culture–technology framework summarized in Figure 1, these measures are intended to bring statutory rule, clinical workflow and patient experience into closer alignment.

These reforms are not only matters of private law. Each carries direct public-health implications. (i) Patient safety: clearer disclosure standards and structured refusal documentation reduce the risk of avoidable harm caused by misunderstood treatment alternatives. (ii) Health equity: a tiered, resource-sensitive technological pathway protects the older adults, illiterate and rural primary-care populations who would otherwise be left behind by uniform digital-consent mandates (30, 31). (iii) System-level trust and dispute burden: aligning legal rule with the clinical encounter is among the most cost-effective ways to reduce defensive medicine, lower litigation volumes and rebuild confidence in the healthcare system (12). Future research should empirically test the proposals advanced here—for example, by evaluating regional pilots of risk-stratified e-consent against measurable outcomes (28), and by surveying patient perspectives on the right to actively seek information (15), on which the existing Chinese empirical literature is still thin. The framework offered above is intended to provide a tractable starting point for that work.
